# Progressive lower extremity cellulitis in a patient with systemic lupus erythematosus on systemic immunosuppression: A rare manifestation of cutaneous tuberculosis

**DOI:** 10.1016/j.jdcr.2024.08.007

**Published:** 2024-08-30

**Authors:** Mariam Alam, Tom Fitzpatrick, Katherine DeNiro, Jason Simmons, Sylvia LaCourse

**Affiliations:** a Department of Dermatology, University of Washington, Seattle, Washington; b Division of Infectious Disease, Department of Internal Medicine, University of Washington, Seattle, Washington

**Keywords:** cellulitis, chronic corticosteroids, cutaneous tuberculosis, immunosuppression, Mycobacterium tuberculosis, tuberculosis, tuberculous cellulitis

## Introduction

Tuberculosis (TB) results from exposure to the *Mycobacterium tuberculosis* (MTB) complex, including MTB and *Mycobacterium bovis* (*M. bovis*). TB most commonly affects the lungs but can involve other organ systems, including, rarely, the skin.^1^

Cutaneous TB has a diverse clinical spectrum that includes both multibacillary cutaneous disease and hypersensitivity reactions to mycobacterial antigens. Multibacillary cutaneous disease can arise from exogenous inoculation (eg, TB chancre, TB verrucosa cutis), contiguous spread (eg, scrofuloderma, orificial TB), or hematogenous dissemination (eg, lupus vulgaris, miliary TB). Here, we present a case of TB cellulitis, a rarely described manifestation of cutaneous TB that typically occurs in patients on systemic immunosuppression.^2^, ^3^, ^4^, ^5^, ^6^, ^7^, ^8^, ^9^

## Case presentation

A 63-year-old woman presented to her primary care clinic with a painful, erythematous plaque on her left anterior shin. Her past medical history was notable for systemic lupus erythematosus treated with prednisone (5 mg/day), hydroxychloroquine, azathioprine, and methotrexate. Other medical conditions included obesity (body mass index 67.9 kg/m2), type 2 diabetes mellitus (hemoglobin A1c 7.1%), and chronic kidney disease.

The patient was originally from northern Mexico and immigrated to the United States 25 years earlier. She had not returned to Mexico nor traveled internationally for many years, and no members of her family had been diagnosed with TB.

At her initial clinic visit (day 0), a solitary erythematous plaque on the left anterior shin measured 11 cm by 15 cm. She denied cough, hemoptysis, weight loss, or night sweats. Duplex ultrasound was negative for deep vein thrombosis. She was prescribed a 10-day course of cephalexin for bacterial cellulitis.

**Fig 1:**
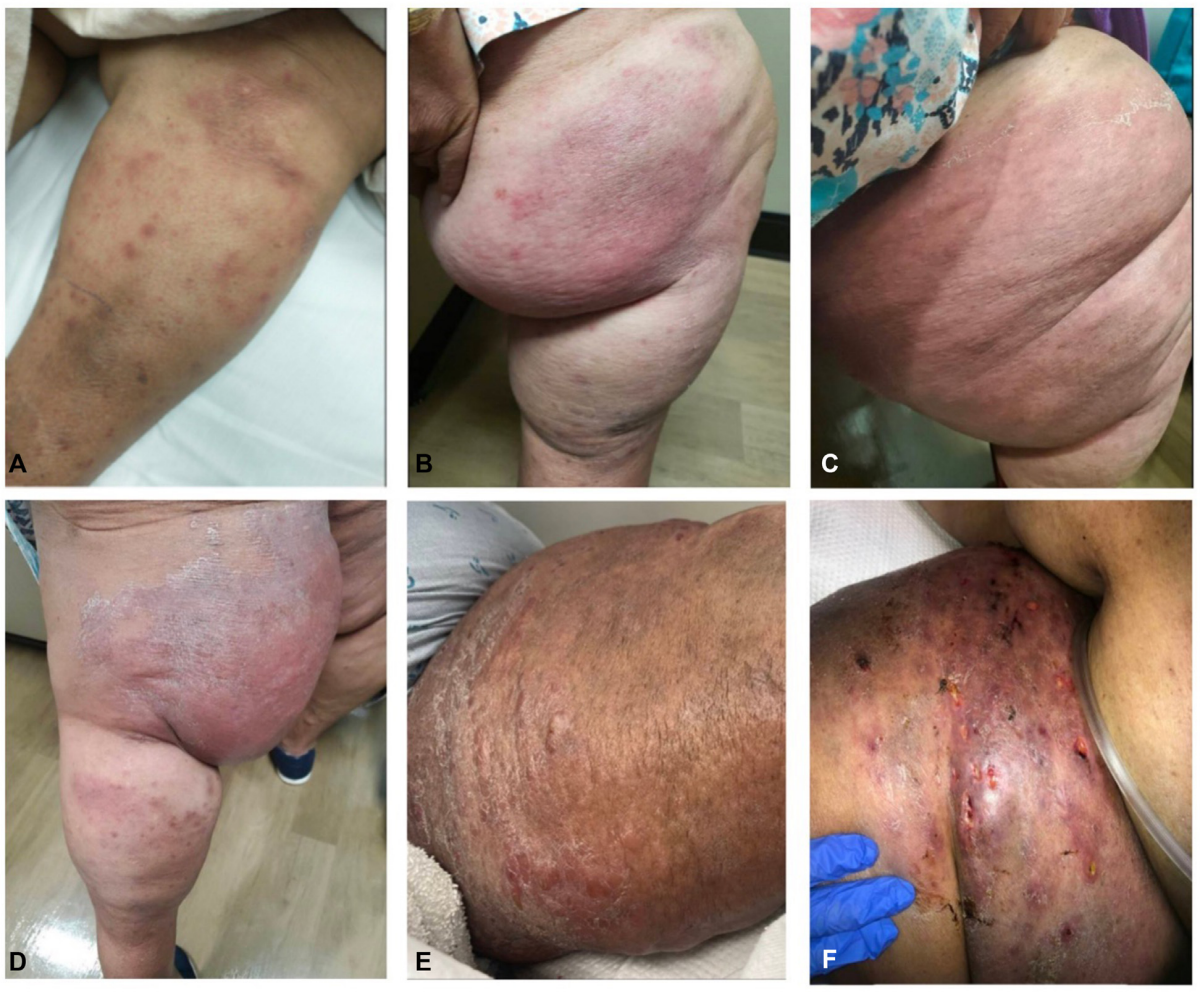
Progression of indurated erythematous plaques on (**A**) left anterior shin on day 12, (**B**) left anteromedial thigh on day 35, (**C**) left anteromedial thigh on day 68, (**D**) left posteromedial thigh on day 68, (**E**) left medial thigh on day 85 (from *side*), and (**F**) left anterior thigh on day 85 (from *above*).

By day 12, the original plaque had expanded, with multiple new plaques diffusely over the anterior left shin (Fig 1, *A*). She was prescribed a 14-day course of doxycycline. Due to concern for vasculitis, prednisone (30 mg/day), azathioprine, and methotrexate were also increased.

Over the subsequent 10 weeks, the plaques on her anterior shin resolved, but new painful erythematous and indurated plaques developed on her anterior and posterior thigh (Fig 1, *B*). These plaques worsened by day 68 (Fig 1, *C* and *D*) despite multiple antibiotics (amoxicillin-clavulanate and clindamycin, vancomycin and ceftriaxone, delafloxacin, and piperacillin-tazobactam) and escalating immunosuppression. Prednisone was increased to 20 mg/day on day 35 and again to 30 mg/12 hours on day 51 due to ongoing concern from her outpatient providers for systemic lupus erythematosus-related rash.

On day 85, she was referred to a tertiary care hospital in Seattle, Washington. Her vital signs were within normal range. Physical examination demonstrated an indurated and edematous erythematous plaque on the proximal left thigh with hyperpigmented nodules extending down the leg at sites of prior involvement (Fig 1, *E* and *F*). Computed tomography of the left lower extremity found non-specific subcutaneous edema with skin thickening.

**Fig 2:**
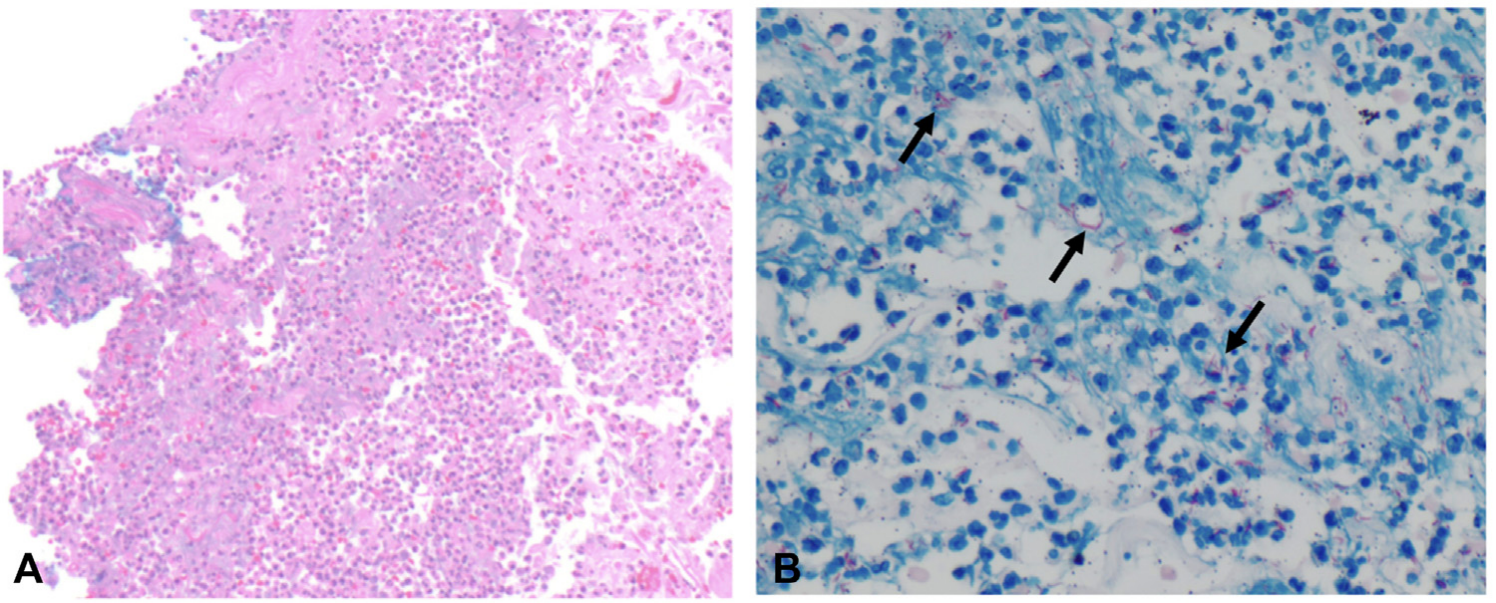
**A,** Hematoxylin and eosin stain showing acanthosis, spongiosis, and neutrophilic microabscesses. **B,** AFB stain with numerous filamentous bacilli (*black arrows*). *AFB*, Acid-fast bacteri.

Punch biopsy of the plaque was performed (day 85). Histopathology showed acanthosis and spongiosis with neutrophilic microabscesses (Fig 2, *A*). Acid-fast bacteria (AFB) stain was positive for innumerable slender filamentous organisms within the dermis (Fig 2, *B*). MTB complex polymerase chain reaction and bacterial polymerase chain reaction panels from skin biopsy tissue were positive for MTB complex. AFB cultures from skin biopsy tissue demonstrated 4+ AFB growth after 6 days, with subsequent susceptibility testing demonstrating isolate susceptibility to all first-line agents. Treatment for cutaneous TB with rifampin, isoniazid, pyrazinamide, and ethambutol was started on day 91, with a plan for 8-week four-drug intensive phase therapy followed by 4-7 months of continuation phase therapy with rifampin and isoniazid.

To evaluate for concurrent pulmonary TB, computed tomography of the chest was obtained, which showed right upper lobe atelectasis and scarring. Sputum AFB stains were negative, but MTB was isolated by culture on solid media after 152 days from 1 of 2 induced sputum samples.

## Discussion

Tuberculous cellulitis is a rare presentation of cutaneous MTB primarily seen in immunosuppressed patients. It presents as an indurated, erythematous plaque that may mimic cellulitis due to typical bacteria or rapidly growing nontuberculous mycobacteria (RGM). Identifying tuberculous cellulitis presents challenges, as evidenced by the delayed diagnosis in this case and a misdiagnosis rate as high as 33% to 50% in some contexts.^1^ Consistent with other cases, our patient’s disease was not limited to the skin, which highlights the importance of a thorough evaluation for distant sites of TB infection.

Most published cases of tuberculous cellulitis occurred in patients on immunosuppressive medications rather than other forms of immunocompromise, such as HIV/AIDS or malignancy (Table I). Besides 1 reported case of tuberculous cellulitis in an immunocompetent patient,^10^ systemic corticosteroids were implicated in all described cases of cutaneous TB.^2^, ^3^, ^4^, ^5^, ^6^, ^7^, ^8^, ^9^, ^11^, ^12^, ^13^ While patients with HIV/AIDS are known to be at elevated risk of other forms of TB, including disseminated TB, tuberculous cellulitis has not been reported in this patient population, perhaps implicating suppression of a CD4^+^-independent pathway in this unique manifestation of cutaneous TB.

**Table I:** Summary of reported cases of tuberculous cellulitis

Study	Clinical history	Morphology	Granulomas present?	Immunosuppressive therapy	Extracutaneous sites of disease
Shigehara et al^3^	85-year-old female with rheumatoid arthritis and methotrexate-lymphoproliferative disorder with a left thigh plaque.	Erythema multiforme-like eruption, followed by painful indurated plaque on left thigh with subsequent ulceration at skin biopsy site dehiscence	No	Methotrexate, tacrolimus; short-term prednisolone (for erythema multiforme-like eruption)	None
Murugesh et al^6^	31-year-old male with renal transplant with right calf erythema.	Tender, erythematous plaque of right foot and calf	Unknown	Prednisolone, mycophenolate mofetil, tacrolimus	None
Rabiei et al^10^	54-year-old healthy male with left-hand erythema.	Pain, erythema, and swelling of left dorsal hand with nonhealing wounds and verrucous lesions	No	None	None
Sakiyama et al^9^	89-year-old male with history of pulmonary TB, polymyalgia rheumatica (PMR), CKD, remote pancreatic cancer with pulmonary nodules, and left thigh plaque.	Erythematous plaque on left thigh	Yes Epithelioid cell granulomas throughout the dermis and subcutis	Prednisolone 5 mg daily	MTB isolated from gastric fluid and sputum cultures
Yeh et al^13^	29-year-old female with Lofgren syndrome with miliary TB and right elbow erythema.	Swelling, erythema of right elbow	Yes Xanthogranulomatous infiltrate	Prednisone	MTB isolated from sputum cultures; imaging consistent with miliary TB
Taguchi et al^4^	67-year-old female with SLE with erythema of right thumb.	Erythematous swelling on the right thumb with erosion and maceration	Yes	Prednisone	None
Koba et al^12^	86-year-old male with bullous pemphigoid, crescenteric glomerulonephritis, with left thigh erythema, found to have miliary TB.	Tender erythematous plaque on left thigh	No	Pulse dose steroids 5 months and again 1 month before presentation, then transitioned to prednisone 20 mg daily.	MTB isolated from sputum cultures; imaging consistent with miliary TB
Kim et al^7^	47-year-old female with dermatomyositis, gastric cancer treated with recent gastrectomy with right axillary plaque, later found to have miliary TB.	Il-defined, tender, erythematous and warm plaque in the right axilla	No	Prednisolone 15 mg daily, intravenous immunoglobulin × 3 sessions	Imaging consistent with miliary TB
Seyahi et al^8^	37-year-old male with renal transplant, chronic hepatitis B, with left calf and foot erythema, found to have miliary TB.	Erythema, swelling of left calf and foot	Yes	Azathioprine 100 mg/day, methylprednisolone 8 mg/day.	MTB isolated from bronchoalveolar lavage cultures, blood cultures, and imaging consistent with miliary TB
Chin et al^11^	28-year-old female with SLE, diffuse proliferative glomerulonephritis with left leg and foot erythema.	Tender, erythematous plaque of the left leg and dorsum of left foot.	Yes	Cyclophosphamide 100 mg × 3 months, prednisolone 60 mg daily decreased to 40 mg daily by time of presentation	None
Lee et al^2^	63-year-old female with DM, chronic steroid use for arthralgia remote history of treated pulmonary TB with left abdomen erythema.	Painful erythema of the left abdomen.	Yes	Chronic steroid use (>20 y)	None
Daido-Horiuchi et al^5^	89-year-old male with CKD, PMR, remote history of untreated pulmonary TB, pancreatic cancer treated with pancreatectomy and splenectomy, gastric cancer treated with endoscopic submucosal dissection, with erythema of left femoral region.	Tender erythema of the left femoral region.	Yes	Prednisolone 5 mg/d × 13 y	Imaging consistent with miliary TB

*CKD*, Chronic kidney disease; *MTB*, mycobacterium tuberculosis; *SLE*, systemic lupus erythematosus; *TB*, tuberculosis.

Previous reports suggest that TB cellulitis can arise from exogenous inoculation, localized contiguous spread, or hematogenous dissemination; however, it is frequently observed in patients with other concurrent foci of TB (Table I). In our case and in several reported cases of TB cellulitis,^7^^,^^8^^,^^12^ skin manifestations preceded diagnosis of pulmonary or miliary TB. Thus, TB cellulitis may herald underlying occult disseminated disease.

Microbiological species obtained from this patient’s skin biopsy showed abundant mycobacteria, which grew in 6 days in liquid broth culture. Culture growth occurs in fewer than half of cases in other forms of cutaneous TB (eg, lupus vulgaris), and when growth occurs, it is typically after more than 3 weeks.^14^ Mycobacterial culture growth within 7 days is classically associated with RGM, which also cause antibiotic-resistant skin and soft tissue infections.^15^^,^^16^ The short time-to-positivity measured in our case likely reflects an especially high bacillary load that developed in the setting of immunosuppression.

This case describes TB cellulitis, a rare cutaneous manifestation of the disease, which in nearly all published reports affects patients on systemic corticosteroids. Broad immunosuppression likely contributes to clinical manifestations that more closely resemble bacterial and RGM infections rather than more common forms of cutaneous TB. Because TB cellulitis clinically resembles typical skin and soft tissue infections, early biopsy should be considered in patients with presumed bacterial cellulitis not responsive to standard antibiotic therapy.

## Conflicts of interest

None disclosed.

## References

[1] Chen Q., Chen W.C., Hao F. (2019). Cutaneous tuberculosis: a great imitator. Clin Dermatol.

[2] Lee N.H., Choi E.H., Lee W.S., Ahn S.K. (2000). Tuberculous cellulitis. Clin Exp Dermatol.

[3] Shigehara Y., Mizuta T., Kasami S. (2022). A case of ulcerative tuberculous cellulitis in the setting of methotrexate-associated lymphoproliferative disorder. JAAD Case Rep.

[4] Taguchi R., Nakanishi T., Imanishi H., Ozawa T., Tsuruta D. (2015). A case of tuberculous cellulitis. Clin Med Insights Case Rep.

[5] Daido-Horiuchi Y., Kikuchi Y., Kobayashi S., Fujii T., Oshima S., Kondo T. (2012). Tuberculous cellulitis in a patient with chronic kidney disease and polymyalgia rheumatica. Intern Med.

[6] Murugesh Anand S., Edwin Fernando M., Srinivasaprasad N.D., Sujit S., Thirumalvalavan K. (2020). Tuberculous myositis and cellulitis in a renal transplant recipient. Indian J Tuberc.

[7] Kim J.E., Ko J.Y., Bae S.C., Ro Y.S. (2011). Tuberculous cellulitis as a manifestation of miliary tuberculosis in a patient with malignancy-associated dermatomyositis. J Am Acad Dermatol.

[8] Seyahi N., Apaydin S., Kahveci A., Mert A., Sariyar M., Erek E. (2005). Cellulitis as a manifestation of miliary tuberculosis in a renal transplant recipient. Transpl Infect Dis.

[9] Sakiyama M., Maeshima H., Chishiki M., Horinosono H., Kawakubo Y. (2016). Tuberculous cellulitis: diseases behind cellulitis like erythema. Cutis.

[10] Rabiei P., Hasanov M., Akhavan B., Aisenberg G.M. (2019). Tuberculous cellulitis in an immunocompetent patient. Proc (Bayl Univ Med Cent).

[11] Chin P., Koh C., Wong K. (1990). Cutaneous tuberculosis mimicking cellulitis in an immunosuppressed patient. Singapor Med J.

[12] Koba S., Okawa T., Misago N. (2013). A case of tuberculous cellulitis lacking epithelioid granuloma. Acta Derm Venereol.

[13] Yeh L.J., Shively N.R., Isacke R.N., Dowling C.A., Stogsdill P.B. (2015). Miliary tuberculosis characterised by lipomembranous fat necrosis. Lancet Infect Dis.

[14] Negi S.S., Basir S.F., Gupta S., Pasha S.T., Khare S., Lal S. (2005). Comparative study of PCR, smear examination and culture for diagnosis of cutaneous tuberculosis. J Commun Dis.

[15] El Helou G., Viola G.M., Hachem R., Han X.Y., Raad I.I. (2013). Rapidly growing mycobacterial bloodstream infections. Lancet Infect Dis.

[16] Gonzalez-Santiago T.M., Drage L.A. (2015). Nontuberculous mycobacteria: skin and soft tissue infections. Dermatol Clin.

